# The developmental profile of temporal binding: From childhood to adulthood

**DOI:** 10.1177/1747021820925075

**Published:** 2020-06-02

**Authors:** Sara Lorimer, Teresa McCormack, Emma Blakey, David A Lagnado, Christoph Hoerl, Emma C Tecwyn, Marc J Buehner

**Affiliations:** 1 School of Psychology, Queen’s University Belfast, Belfast, UK; 2 Department of Psychology, The University of Sheffield, Sheffield, UK; 3 Department of Psychology, University College London, London, UK; 4 Department of Philosophy, The University of Warwick, Coventry, UK; 5 School of Social Sciences, Birmingham City University, Birmingham, UK; 6 School of Psychology, Cardiff University, Cardiff, UK

**Keywords:** Temporal binding, causal binding, causality, intentional action, time perception, intentional binding

## Abstract

Temporal binding refers to a phenomenon whereby the time interval between a cause and its effect is perceived as shorter than the same interval separating two unrelated events. We examined the developmental profile of this phenomenon by comparing the performance of groups of children (aged 6–7, 7–8, and 9–10 years) and adults on a novel interval estimation task. In Experiment 1, participants made judgements about the time interval between (a) their button press and a rocket launch, and (b) a non-causal predictive signal and rocket launch. In Experiment 2, an additional causal condition was included in which participants made judgements about the interval between an experimenter’s button press and the launch of a rocket. Temporal binding was demonstrated consistently and did not change in magnitude with age: estimates of delay were shorter in causal contexts for both adults and children. In addition, the magnitude of the binding effect was greater when participants themselves were the cause of an outcome compared with when they were mere spectators. This suggests that although causality underlies the binding effect, intentional action may modulate its magnitude. Again, this was true of both adults and children. Taken together, these results are the first to suggest that the binding effect is present and developmentally constant from childhood into adulthood.

The relation between time and causality in adults is bidirectional: not only is temporal information used when making causal inferences (e.g., Bramley et al., 2018; Shanks, Pearson & Dickinson, 1989), but causal representations influence the perception of both the temporal order of and temporal interval between events (Bechlivanidis & Lagnado, 2013, 2016; Buehner, 2012, 2015; Haggard et al., 2002; Tecwyn et al., 2020). The perception of a cause and its direct effect as temporally closer than two causally unrelated events is known as *temporal binding*, and it is this phenomenon, and specifically its developmental profile, that is the focus of the current study.

Although initial research suggested that temporal binding was primarily observed in contexts in which the cause is an intentional action (e.g., a button press that causes a tone, Haggard et al., 2002), subsequent studies indicate that this phenomenon generalises to other sorts of causal–effect relations (Buehner, 2012; Suzuki et al., 2019). Considerable research in the last two decades has examined the nature of temporal binding (e.g., Buehner & Humphreys, 2009; Engbert et al., 2008), what factors modulate its magnitude (e.g., Moreton et al., 2017; Poonian & Cunnington, 2013), and how it presents in clinical populations (Haggard et al., 2003; Voss et al., 2010). However, as yet, its developmental profile is unclear. To date, only three studies have explored temporal binding in children, and, moreover, their findings are inconsistent. Thus, it is not known to what extent causal representations have similar top-down effects on time perception in children as in adults.

Cavazzana et al. (2014) were the first researchers to study temporal binding in children. In their study, 8- to 10-year-olds and adults watched a screen as a series of letters flashed up in quick succession. Participants had to report which letter was on the screen when a target event occurred. The target events to be judged (i.e., for which concurrent letters were to be reported) included a voluntary button press that caused a tone, the occurrence of a tone that was followed by another tone, or the tone that followed either of these first events. This novel paradigm produced results typical of temporal binding in adults: the voluntary action and tone were judged as occurring closer together in time than two causally unrelated tones. However, this pattern was not observed in children. Cavazzana et al. (2017) subsequently reported similar findings using the same paradigm, and argued that difficulties in attentional control may account for the lack of temporal binding in children. They suggested that children were not able to direct their attention to the critical target events as they were instead distracted by peripheral events. However, this leaves open the possibility that temporal binding might be observed in children in a paradigm that does not place excessive demands on attentional resources, which are known to be underdeveloped in children (see Anderson, 2002 for review).

Indeed, more recently, Blakey et al. (2019) have reported evidence of binding in children considerably younger than those studied by Cavazzana et al. (2014, 2017). Blakey et al. (2019) used a simpler task in which participants anticipated when an event would occur, rather than retrospectively reporting the perceived time of an event’s occurrence. In the study, 4- to 11-year-olds completed a stimulus anticipation task in which they pressed a button to indicate when they believed a target event (the launching of a rocket on a computer screen) was going to occur. Their first experiment compared a self-causal condition in which children pressed a button that caused the rocket to launch following a delay with a non-causal condition in which the rocket launched following a delay after a predictive signal. Their second experiment also included a machine–causal condition in which a mechanical lever pressed a button that caused the rocket to launch following a delay. Participants of all ages responded in a more anticipatory manner in the causal conditions. That is, they expected the outcome of causal button presses to occur earlier than outcomes that followed a non-causal predictive signal. These results provided the first evidence that children’s causal representations influence their perception of time, with the authors arguing that temporal binding reflects a fundamental and early-developing way in which causal cognition and temporal perception interact.

Blakey et al.’s (2019) findings indicate that children’s as well as adults’ temporal perception is affected by causal representations; what remains unclear is whether the extent of this influence is developmentally stable from childhood into adulthood. Making child–adult comparisons is difficult using existing paradigms. As discussed previously, Cavazzana et al.’s (2014, 2017) task may be too cognitive demanding for children. On the contrary, the paradigm used by Blakey et al. (2019), though more child-friendly than that of Cavazzana et al., also has its shortcomings. Specifically, Droit-Volet (2010) strongly advises against using motor-dependent tasks, such as the stimulus anticipation task of Blakey et al. (2019), when comparing the temporal perceptual abilities of adults and children because children typically take longer to initiate and complete movements than adults.

## The current study

The goal of the current study was to establish a developmental profile for the temporal binding effect across childhood and into adulthood, resolving existing inconsistencies in the literature. Because assessing temporal binding involves comparison of a causal and non-causal condition, the task needed to be set in context that allowed for causal and non-causal event pairings; we adopted Blakey et al.’s (2019) rocket launching scenario for this purpose. However, to address the methodological issues that have been described, we measured time judgements differently. This involved developing a novel paradigm suitable for assessing time perception in both adults and children. Specifically, we sought to devise a paradigm sufficiently sensitive to detect the well-established developmental effects that have been shown to exist within the time perception literature (e.g., Block et al., 1999; Droit-Volet et al., 2001; McCormack et al., 1999), without placing excessive demand on attention or motor control abilities.

To this end, we devised a categorical interval estimation task that had some structural resemblances to tasks previously used to examine time perception in children (Droit-Volet & Izaute, 2009; Droit-Volet & Wearden, 2001) but also to tasks used to measure temporal binding in adults (Ebert & Wegner, 2010; Kumar & Srinivasan, 2017; Wen et al., 2015). Participants were initially trained to identify four intervals of different lengths (the categories). At test, participants then reported the time interval between two events by judging which category the interval matched. Participants completed both a causal condition and a non-causal condition; see Figure 2. In the causal condition, participants pressed a button that caused a rocket to launch following a delay. In the non-causal condition, participants simply observed a predictive signal that indicated the rocket would launch after a delay. Participants gave an estimate of the time interval between the button press (causal condition) or predictive signal (non-causal condition), and the rocket launch, by choosing the category that matched the interval. The index of temporal binding was whether participants judged intervals to be shorter in the causal condition compared with the non-causal condition.

Even young children can produce meaningful data in simple categorical timing tasks that involve two time intervals: in the temporal bisection task participants are exposed to “short” and the “long” reference durations, and then judge whether other intervals are more similar to the short and long references (e.g., Droit-Volet et al., 2007; Droit-Volet & Wearden, 2001; Zélanti & Droit-Volet, 2011). However, we were concerned that the bisection task, with its use of just two categories, would not be sufficiently sensitive to pick up binding effects, which are typically small and of the order of tens of milliseconds. Indeed, previous research suggests that the bisection task does not reliably pick up age differences between middle childhood and adulthood (e.g., Droit-Volet et al., 2004; McCormack et al., 1999), which may reflect a lack of sensitivity. Moreover, although categorical timing tasks with multiple categories have been used to successfully demonstrate temporal binding with adults (Ebert & Wegner, 2010; Kumar & Srinivasan, 2017), there are no published studies that have used the bisection task. While the current task used fewer categories than those used with adults (4 rather than 10), initial pilot work with adults indicated that it was sufficiently sensitive to allow measurement of temporal binding. However, the use of four response options and the associated instructions meant that the task was too difficult for preschoolers. Thus, our youngest age-group was 6- to 7-year-olds; we also included two older groups of children and an adult group.

## Developmental predictions

Given the limited number of studies that have explored the binding effect in children, and their conflicting results, it is difficult to confidently generate predictions concerning the developmental profile of the binding effect across this age range. Temporal binding can be seen as a top-down effect of causal beliefs on time perception, raising the possibility that this effect emerges or strengthens developmentally as children gain experience with the causal structure of the world. However, the work of Blakey et al. (2019) suggests that even preschoolers’ time perception is susceptible to influence from their causal representations. Moreover, Tecwyn et al. (2020) have demonstrated that the causal representations of 4- to 10-year-old children influence their judgements about the temporal order of events in a similar way to adults. That is, children reorder events to align with their causal beliefs in the same way as adults do (Bechlivanidis & Lagnado, 2013, 2016). The relation between temporal binding and this type of reordering effect is poorly understood, and it remains unclear whether the same mechanisms underpin the two effects. Nevertheless, Tecwyn et al.’s findings suggest that the bidirectional relationship between time and causality is developmentally stable, at least from 4 years of age. It is therefore possible that the magnitude of temporal binding effects will not differ across our age-groups.

Alternatively, children may demonstrate *greater* binding than adults. To a greater extent than adults, children favour temporal cues over other sources of information when determining causal structure (McCormack et al., 2015), even when a temporally distal candidate cause is statistically more likely (Siegler & Liebert, 1974), or a longer delay is compatible with mechanism information (Schlottmann, 1999). Taken together, these findings suggest a particularly close relation between temporal and causal cognition in children, with children placing greater weight on temporal cues when making causal judgements. This raises the possibility that children’s causal representations may also have a stronger effect on their perception of temporal intervals, that is, that children may show greater binding than their adult counterparts. Indeed, such bidirectional strong links between time and causation early in development could potentially support acquisition of stable causal beliefs. That is, temporal contiguity may serve a simple heuristic that typically yields causal beliefs, but once an initial belief is formed, it may in turn be reinforced as a result of temporal binding exaggerating the temporal proximity of causes and their effects.

## Experiment 1

### Method

#### Participants

A total of 142 participants completed the task: 40 6- to 7-year-olds (*M_age_* = 82 months, *SD_age_* = 3.60, range: 73–88 months, 35% female), 31 7- to 8-year-olds (*M_age_* = 100 months, *SD_age_* = 3.79, range = 90–106 months, 50% female), 37 9- to 10-year-olds (*M_age_* = 126 months, *SD_age_* = 3.63, range: 119–131 months, 30% female), and 34 adults (*M_age_* = 278 months, *SD_age_* = 82.8 months, range: 218–523 months, 76% female). The child participants were recruited from three different school-year groups. Adult participants were undergraduate students participating in exchange for course credit. Ethical approval was granted by the Research Ethics Committee of the university of the first author.

#### Materials

The experiment was completed by participants individually in a quiet area. Participants sat in front of a Dell laptop computer (60 Hz refresh rate) with a 15.6 in. screen, to which a 4-button Black Box Toolkit USB response box was connected. The experiment was run using EPrime 2.0 software (Psychology Software Tools, 2016).

#### Design and procedure

**Figure 1.**
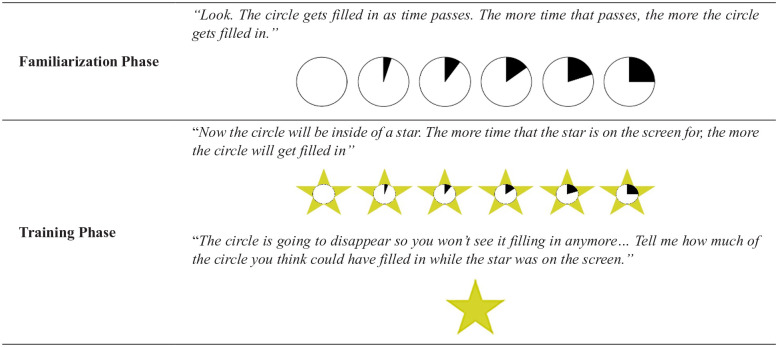
Overview of the first two task phases. Participants were trained to associate the circle segments with different amounts of time.

**Figure 2.**
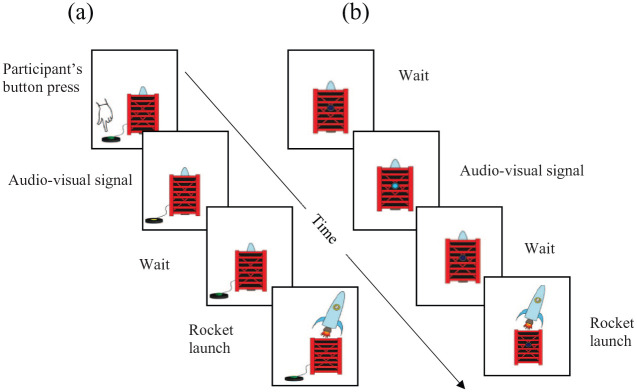
Schematic of the (a) causal and (b) non-causal conditions of the experimental phase.

The task comprised three phases. The first two phases were designed to introduce participants to the equipment and task features that would enable them to complete the third phase with accuracy. Figure 1 provides an overview of the first two phases; Figure 2 outlines the final, experimental phase.

##### Familiarisation phase

Participants were first shown a demonstration of how a circle “fills in” as time passes. Specifically, they were shown that it took “a little bit of time” (200 ms) for one-fourth of the circle to fill in, a “bit more time” (400 ms) for half of the circle to fill in, “even more time” (600 ms) for three-fourths of the circle to fill in, and finally it took the “most time” (800 ms) for the full circle to be filled in completely. The appearance of the circle “filling in” was created by showing a series of images in quick succession.

Participants’ ability to correctly match an onscreen circle segment to the corresponding segment on a button box was assessed in a series of trials. To do this, they watched as a circle appeared onscreen, began filling in, and then disappeared, after which they were asked, “How much of the circle filled in that time?” Feedback was provided at the end of every trial. The task moved on after four correct responses in a row or after 12 trials.

##### Training phase

Next, participants completed a temporal training phase in which they learned to associate each of the four circle segments with a specific delay. Following this, they were tested on their ability to accurately identify each of the target delays. The aim of this phase was to enable participants to accurately use the circle segments as a proxy for an estimate of time. Participants saw the circle embedded within a star and were told that the longer the star stayed on screen for, the more of the circle would get filled in. Participants watched as the circle in the middle of the star filled in while it was onscreen. Participants used the response box to indicate how much of the circle had filled in while the star was on the screen.

Participants were then told that the circle was going to disappear behind the star so they could no longer see it filling in. They then completed a series of trials in which a star flashed up on the screen and stayed there for one of the four target delays (200, 400, 600, and 800 ms) before disappearing. After the star had disappeared from the screen participants were asked, “How much of the circle could have filled in while the star was on the screen?” Feedback was provided at the end of every trial. Delay order was randomised across trials. The training phase ended when participants got four answers correct in a row or when they completed 40 trials, whichever came first. Prior to analysis, those participants who did not achieve four correct answers in a row were excluded. Table 1 shows the average number of training trials per age-group.

**Table 1.** Average number of training phase trials (*SD*) per age-group.

Age-group	Experiment 1	Experiment 2
6–7-year-olds	8.40 (3.69)	8.66 (3.81)
7–8-year-olds	9.96 (5.09)	8.97 (4.71)
8–9-year-olds	N/a	8.73 (3.93)
9–10-year-olds	8.59 (3.92)	9.00 (5.04)
Adults	6.88 (2.01)	7.48 (3.53)

##### Experimental phase

The experimental phase consisted of two conditions, one causal (Figure 2a) and other non-causal (Figure 2b). In this portion of the task, participants used their newly acquired understanding of the delays and associated circle segments to estimate the time between two events. In the causal condition, participants used the response box to indicate the length of time between a tone that accompanied their button press and a subsequent rocket launch. In the non-causal condition, they estimated the time between a predictive signal and rocket launch. The two conditions of the experimental phase were blocked so that all trials in one condition were completed before the next condition started. Both conditions were completed by all participants, in counterbalanced order.

In the causal condition, participants were told that a rocket would “start getting ready to launch” when they pressed the launch button that was in front of them. Their button press was accompanied by a “beep” and visual of the onscreen button depressing. In the non-causal condition, the rocket started getting ready from a signal consisting of an onscreen flash and an audible beep. When the rocket launched, a “whoosh” was heard and the onscreen rocket moved to the launched position. After each launch, participants indicated how much of the circle they thought would have filled in while the rocket was “getting ready.” In both conditions, the time the rocket spent “getting ready” was the time between the audiovisual signal, and the “whoosh” that accompanied the rocket moving to the launched position. Participants were instructed that this was the interval to be judged. The delay between the first event (the button press, or the predictive signal) and the rocket launch was 300, 500, or 700 ms. The delay was randomised with eight presentations of each delay in each condition, making 24 trials in each condition and 48 trials in total. The participants were naïve to the fact that the delays were not the same as those in the training phase. In using delays in the experimental phase that fell between those learnt in the previous two phases, participants were unknowingly forced to choose whether they experienced the experimental delays as more similar to intervals that were slightly longer or shorter than they were in reality. Temporal causal binding would thus manifest as a higher probability to choose a shorter interval in causal compared with non-causal conditions.

### Results

**Figure 3.**
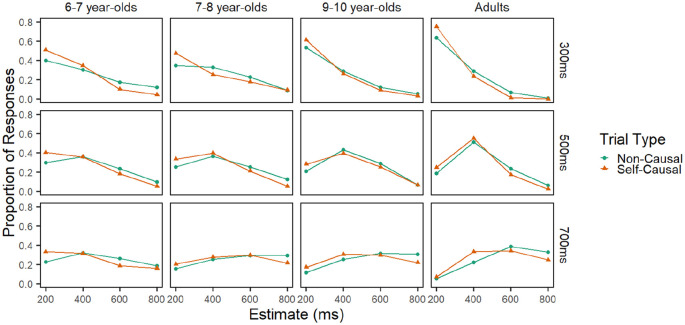
Proportion of each response type for Experiment 1 panelled by age-group (horizontally) and delay (vertically).

Only the data of those participants who passed the training phase were analysed. This criterion excluded 19 participants from analysis: 10 6- to 7-year-olds, five 7- to 8-year-olds, and three 9- to 10-year-olds. This left 34 adults, 34 9- to 10-year-olds, 26 7- to 8-year-olds, and 30 6- to 7-year-olds in the analysis. The proportion of times participants selected each response category (200, 400, 600, and 800 ms) for each of the three target delays can be seen in Figure 3, as a function of delay, condition, and age-group.

The ordinal package in R (Christensen, 2018; R Core Team, 2014) was used to perform a cumulative link mixed model analysis of participants’ responses. A backward elimination approach was taken, in that a full model, encompassing condition, delay, and age as factors, was fitted before the model was reduced by eliminating non-significant factors. Participant ID was included as a random effect to account for the repeated-measures nature of the design as well as individual differences in response scale use. Variables were dummy-coded such that the causal condition and the adult age-group were used as reference categories. Delay was set as an ordinal level variable meaning no reference category was required; instead, the model explores changes in the outcome variable that arise with each increase in delay (for example, when increasing from 300 to 500 ms and from 500 to 700 ms). The Akaike information criterion (AIC) was used as a method of assessing goodness of fit, where lower AIC values represent a better fitting model (Schermelleh-Engel et al., 2003).

The results of the final model can be seen in Table 2. It is important to note here that although the data shown in Figure 3 are on their observable scale (i.e., proportion of each response type given), the modelling is of log odds because this latent scale is considered more appropriate (Dixon, 2008). Briefly, the results indicate that participants’ estimate of delay increased significantly with each increase in the target delay, indicating a sensitivity to the manipulation of delay. Although all age-groups showed an ability to give higher estimates in response to greater delays, the likelihood of this happening increased with age. Crucially, participants were more likely to give higher estimates of delay in the non-causal condition compared with the causal condition, demonstrating temporal binding.

**Table 2.** Results of cumulative link mixed model.

Parameter	β (*SE*)	Odds ratio	*z*
Condition			
Non-causal	0.45 (.051)	1.57	8.82***
Delay (ms)			
Ascending	2.36 (.10)	10.6	23.9***
Delay (ms) × age-group (years old)			
Delay × 6–7	−1.64 (.13)	0.19	−12.6***
Delay × 7–8	−1.37 (.13)	0.25	−10.1***
Delay × 9–10	−.68 (.13)	0.51	−5.34***

Note: The original model consisted of the following terms: condition, delay, age-group, condition × delay, condition × age-group, delay × age-group, condition × delay × age-group, which gave an AIC value of 13,306. This model was then reduced to include only the significant terms shown in Table 2, which gave an improved AIC value of 13,292 suggesting that the final model is a better fit for the data. Reference categories used were causal-condition and adult age-group. All effects were significant at the ***p < .001 level.

#### Delay effects

The positive main effect of delay indicates that participants were more likely to give higher estimates for higher delays. The significant interaction between delay and age-group indicates that there was a developmental change in the ability to accurately discriminate between the target delays. Inspection of the beta values and odds ratios indicates that the odds of the youngest age group giving a higher response to longer delays were less than the odds of adults doing so. This difference decreases with age, although even the older children were less accurate than the adults. This suggests that children’s ability to discriminate between the delays increases with age throughout childhood and into adulthood. The above model was rerun with each age-group as the reference category in turn. This allowed for more thorough comparisons of the developmental age effects. The results indicated that the effect of delay in each age-group was significantly different from all other age-groups; younger age-groups were less likely than older age-groups to give higher estimates in response to greater delays (all *p* < .05). This indicates that the task is appropriately sensitive to detect developmental changes in time perception. To ensure that participants of every age-group could accurately discriminate between the target delays, that is, appropriately engage with the task, the age groups were considered independently, and response data was modelled with delay as a predictor variable and participant as a random factor. The results indicate that delay was a significant, positive predictor of response in every age-group, all *p*s < .001.

#### Effect of condition

Critical to the aim of the study, the results indicate that response varied significantly as a factor of condition. As can be seen in the “Odds ratio” column in Table 2, participants were significantly more likely to give a higher temporal estimate in the non-causal condition than the causal condition, independent of age and delay. This pattern of results is typical of the temporal binding effect in which participants perceive delays in causal contexts as shorter than delays in non-causal contexts. Condition did not interact significantly with age.

### Discussion

The results indicate that both children and adults perceive the temporal interval between a cause and its effect as shorter than the interval between a non-causal signal and subsequent event. Although children were less accurate in the timing task than adults, the magnitude of the binding effect did not differ with age. These results provide the first evidence that the binding effect, previously only observed in children and adults in separate experimental paradigms, is developmentally stable, at least from 6 years of age. These results extend those of Blakey et al. (2019) by showing that causal representations influence the time perception of both children and adults in the same way and to the same extent. Although previous research has suggested that temporal information is weighted more heavily in young children’s determination of causal structure than adults’ (e.g., McCormack et al., 2015; Schlottmann, 1999), there was no evidence that causal beliefs had a greater influence on children’s duration judgements; the causal representations of children appear to affect their experience and perception of time in much the same way as adults.

Not only is the task presented in Experiment 1 the first task that allows for binding to be explored in both adults and children, but the task itself is a novel way of assessing time perception in children. The results indicate that young children differentiated between the delays to a lesser extent than older children and adults. This is in line with many previous developmental time perception studies (see Droit-Volet, 2003 for review) that show that children’s temporal discrimination ability is less precise than adults’ (e.g., Droit-Volet, Clément & Fayol, 2003; Droit-Volet & Wearden, 2001; McCormack et al., 1999). That being said, these previous developmental patterns have been observed only in *explicit* timing tasks in which participants were overtly instructed to attend to the delay between events. When participants complete *implicit* timing tasks, in which attention is not drawn to the temporal features of events, similar age-related variation is not observed (Coull & Droit-Volet, 2018; Droit-Volet & Coull, 2016). Nevertheless, our developmental time perception results compare well with those using well-established, explicit measures.

However, a higher number of the youngest children (6- to 7-year-olds) failed the temporal training relative to the other age-groups, and the proportion of participants who passed the training phase improved with age. This is not wholly unexpected given that the paradigm is more complicated than classic timing tasks, such as the generalisation or bisection tasks (e.g., Droit-Volet, 2003; Droit-Volet & Izaute, 2005; Lustig & Meck, 2011; McCormack et al., 1999) that only require participants to remember one or two reference durations. Importantly though, the majority of even the youngest children successfully completed the time training phase and showed a sensitivity to delay in the test phase, indicating that they understood and remembered the mapping of temporal durations onto the circle segments that were used as a proxy for their time estimates. Thus, the task developed in Experiment 1 has the potential to be used as a novel way of assessing developmental differences in time perception beyond the binding effect.

## Experiment 2

There is some debate within the temporal binding literature concerning what underlies the effect. Originally, researchers thought that binding occurs *only* for intentional action (i.e., actions one has deliberately carried out oneself; Haggard et al., 2002). However, subsequent research has shown that causality, irrespective of intentionality, is both necessary (Buehner & Humphreys, 2009) and sufficient (Buehner, 2012) to bring about the binding effect in adults. Similarly, Blakey et al. (2019) found that the magnitude of the binding effect in children did not vary significantly as a product of who or what (self or machine) initiates the causal action, suggesting that it is the presence of causality that is critical. With these previous findings in mind, we have assumed thus far that causality rather than intentionality of action drives the binding effect observed in Experiment 1.

However, some past research with adults has shown that intentionality may modulate the magnitude of the binding effect in adults, with the effect being greater when causes are self-generated actions rather than the observed actions of another person (e.g., Buehner, 2012; Dogge et al., 2012). Given that Blakey et al. (2019) found no such evidence of a bolstered effect in the context of self-generated action in their 4- to 11-year-old participants, this modulation of the effect may be specific to adults. That being said, other studies with adults have found no such evidence of a bolstered effect in self-causal contexts (Poonian et al., 2015; Suzuki et al., 2019). This may indicate that, rather than being a developmental trend in the effect, the modulation of the binding effect through the addition of intentional action may instead be task-dependent.

Experiment 2 sought to address this issue by adapting our categorical timing task to explore the binding effect in two causal conditions: one in which the cause of the rocket launch was the participant’s own intentional action, and the other in which the cause was the experimenter’s action. Of interest was whether the binding effect was greater for self-causal trials compared with other-causal trials, and whether any modulating effect of intentional action was developmentally constant.

### Method

#### Participants

A total of 110 participants took part in the experiment: 33 6- to 7-year-olds (*M_age_* *=* 88 months, *SD_age_* = 3.62, range: 82–94, 64% female), 30 7- to 8-year-olds (*M_age_* *=* 101, *SD_age_* = 3.12, range: 95–107, 40% female), 24 8- to 9-year-olds (*M_age_* _*=*_ 113 months, *SD_age_* = 3.99, range: 105–118, 50% female), 27 9- to 10-year-olds (*M_age_* *=* 124 months, *SD_age_* = 3.29, range: 119–130, 48% female), and 29 adults (*M_age_* = 275 months, *SD_age_* = 71.4, range: 223–529, 86% female). The child participants were recruited from three different school-year groups. Adult participants were undergraduate students participating in exchange for course credit.

#### Design and procedure

The method employed was the same as that of Experiment 1, except with an additional condition in the experimental phase. The added experimenter–causal condition consisted of a block of 24 trials, 8 of each target delay, just as with the self-causal and non-causal conditions, giving 72 trials in total. In this condition, participants were required to watch the experimenter press the button, which resulted in a rocket launch following one of three target delays.

The experimenter pressed the button at a random time after the start of each trial, ensuring the participant was focused on the task before doing so. The experimenter based the timing of their button presses on the average latency of participants’ button presses from Experiment 1, taking into account the age of the participant. Generally, the experimenter’s button press occurred within the first 2,000 ms of the start of the trial. The experimenter’s button press was accompanied by an audible beep just as the participant’s was. This ensured that the participant was aware that the button had been pressed and that the interval to be judged had started. Just as with the other two conditions, after every trial the participants were asked how much of the circle they thought could have filled in while the rocket was “getting ready to launch.”

### Results

**Figure 4.**
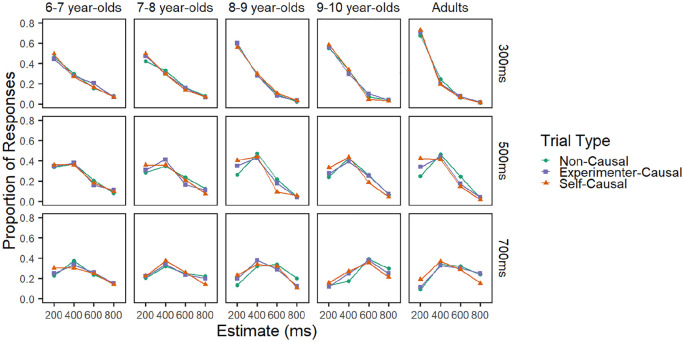
Proportion of each response type in Experiment 2 panelled by age-group (horizontally) and delay (vertically).

As with Experiment 1, only the data of those participants who passed the training phase with four-in-a-row correct were analysed. This criterion excluded four participants from analysis: one 6- to 7-year-old, two 8- to 9-year-olds, and one 9- to 10-year-old. This left 32 6- to 7-year-olds, 30 7- to 8-year-olds, 22 8- to 9-year-olds, 26 9- to 10-year-olds, and 29 adults in the final dataset. As with Experiment 1, the proportion of each response (200, 400, 600, and 800 ms) to each of the three delays was calculated for the three conditions. These data can be seen in Figure 4.

Again, as with Experiment 1, the response data of Experiment 2 were analysed using R’s ordinal package (Christensen, 2018; R Core Team, 2014). The same backward elimination approach taken in Experiment 1 was once again employed. Variables were dummy coded such that the non-causal baseline condition, and adult age-group were used as reference categories. The final model can be seen in Table 3.

**Table 3.** Results of cumulative link mixed model.

Parameter	β (*SE*)	Odds ratio	*z*
Condition			
Self-causal	−0.37 (.047)	0.69	−7.82***
Experimenter-causal	−0.14 (.047)	0.87	−2.90**
Delay (ms)			
Ascending	2.08 (.080)	8.00	26.0***
Delay (ms) × age-group (years old)			
Delay × 6–7	−1.34 (.11)	0.26	−12.7***
Delay × 7–8	−1.12 (.11)	0.33	−10.5***
Delay × 8–9	−0.66 (.12)	0.68	−5.63***
Delay × 9–10	−0.23 (.11)	0.79	−2.10*

Note: The original model consisted of the following terms: condition, delay, age-group, condition × delay, condition × age-group, delay × age-group, condition × delay × age-group, which gave an AIC value of 22,982. This model was then reduced to include only the significant terms shown in Table 3, which gave an improved AIC value of 22,960 suggesting that the final model is a better fit for the data. Reference categories used were non-causal condition and adult age-group.

Significance codes: *p < .05. **p < .01. ***p < .001.

#### Delay effects

The positive main effect of delay indicates that participants were more likely to give higher estimates for higher delays. The interaction between delay and age-group indicates that the ability to discriminate between delays varied with age. The results indicate that children of all ages were significantly less likely than adults to give a higher estimate in response to longer delays. The ability to discriminate between delays increased with age. The above model was rerun with each age-group as the reference category in turn. This allowed for more thorough comparisons of the developmental age effects. The results indicated that the effect of delay in each age-group differed significantly from all other age-groups with younger age-groups being less likely than older age-groups to give higher estimates in response to greater delays (all *p* < .05). As with Experiment 1, the data were split by age-group and the analysis was rerun to ensure that participants of every age-group could accurately discriminate between the target delays. The results indicate that delay was a significant and positive predictor of response in every age-group, all *p*s < .001.

#### Effect of condition

Critical to the aims of the study, participants were more likely to give higher temporal estimates in the non-causal condition than in either the self-causal or experimenter-causal conditions. This indicates that participants experienced delays as shorter in the two causal conditions than in the non-causal baseline, a pattern of results typical of temporal binding. Pairwise comparisons were run to explore the difference between the two causal conditions. Results reveal that participants were more likely to give a higher temporal estimate in the experimenter–causal condition than in the self-causal condition, β (*SE*) = .23 (.047), odds ratio = 1.26, *p* < .001. These results suggest that the presence of intentional action bolsters the magnitude of the binding effect.

### Discussion

The results of Experiment 2 showed that participants of all ages were more likely to perceive delays between causally related events as shorter than the same delay between two unrelated events. These results replicate the findings of Experiment 1 in a new sample. In addition, the results suggest that the magnitude of binding is greater for self-generated action–outcome sequences compared with observed action–outcome sequences. This latter result suggests that the addition of intentional action bolsters the binding effect. The lack of a developmental trend in these results further indicates that the binding effect is present and consistent from at least 6 years of age into adulthood.

The finding that the binding effect was stronger for self-generated action–outcome sequences than observed sequences is consistent with some past studies that have used adult participants (e.g., Buehner, 2012; Dogge et al., 2012). However, it contrasts with the findings of Blakey et al. (2019), who observed binding of equal magnitude in children both when they caused the outcome themselves and when a machine caused the outcome. This disparity in results may be indicative of task-related differences in how the binding effect presents. Indeed, even studies with adults on whether the addition of intentionality alters the effect have produced mixed results (e.g., Poonian et al., 2015; Suzuki et al., 2019). Why this is the case is not clear, highlighting the fact that although the binding effect has been consistently found using many different types of timing tasks, the mechanism or mechanisms underpinning this effect are still not fully understood.

## General discussion

In this study we developed a novel, child-friendly paradigm to measure temporal binding. We were able to compare the binding effect in both adults and children for the first time. The results showed that both adults and children were more likely to perceive delays between cause and effect as shorter than the same delay between a predictive signal and outcome. The results of Experiment 1 provide support for the notion that the binding effect is not a late-emerging phenomenon; rather it is observable and consistent from at least 6 years of age. Experiment 2 replicated the results of Experiment 1, and extended them by showing that although the binding effect is observable in causal contexts in which the participants’ intentional action is not the cause of an outcome, the presence of intentional action bolsters the magnitude of the effect. Again, this was true of both adults and children.

Arguably, the method developed and utilised here to assess binding is the first experimental paradigm that is suitable for studying binding in participants across a wide age-range. We have suggested that the two experimental paradigms used within this area in the past have either not been ideal for use with child participants (i.e., Cavazzana et al., 2014, 2017), or been unsuitable for comparisons between children and adults (i.e., Blakey et al., 2019). Our results indicate that the task we used, which required minimal motor skills and was less cognitively demanding than that of Cavazzana et al., was suitable for adults and children. Perhaps unsurprisingly, there was age-related variation in participants’ temporal discrimination in both experiments. Children were less accurate than adults in discriminating between the test durations, and this was particularly true of the youngest group of children. This aligns well with previous research that has shown a general age-related improvement in the accuracy of time perception (Droit-Volet et al., 2003; Droit-Volet & Izaute, 2009; McCormack et al., 1999). Importantly, even though timing improved in accuracy developmentally, participants of every age discriminated between the target delays, suggesting that the task is sufficiently sensitive for use with both children and adults. This task yielded evidence of binding in all age-groups indicating that, contrary to the claims of Cavazzana et al. (2014, 2017), the binding effect is not late-emerging as long an age-appropriate paradigm is used.

The method used here to assess children’s ability to make sub-second timing judgements may be of interest to those exploring binding—and timing more generally—in both child and adult populations. One of the benefits of this paradigm is that it does not require knowledge or use of conventional timing units. This is not only advantageous for use with children, whose knowledge of clock units is limited (see Block et al., 1999 for review), it also has its advantages for use with adult populations. For example, using a categorization judgement allows for the unusual task of explicitly quantifying a sub-second temporal interval (a method used in some studies of binding) to be circumvented. Although keeping track of sub-second intervals is essential for the completion of everyday tasks, explicitly quantifying temporal intervals of such small magnitude is something that is typically only performed during experimental tasks in lab settings.

Our categorization task has some similarities with the temporal bisection task that is used extensively in timing studies, including many with children (e.g., Droit-Volet & Wearden, 2001; McCormack et al., 1999). As mentioned in the “Introduction”, we did not use a bisection task because we were concerned about the sensitivity of such a task with regard to measuring binding. Use of a more complex categorization task had a further advantage, which is that it allowed us to explore whether binding was present across a set of different target delays (300, 500, and 700 ms). The delays at which the binding effect is observable have been found to vary both within and between paradigms (e.g., Berberian et al., 2012; Buehner & Humphreys, 2009; Haggard et al., 2002). Our analysis did not find that the magnitude of the binding effect varied with delay length, indicating that in the current paradigm, at least across this range of delays, the effect is robust.

The results of both experiments also indicated that there is no developmental variation in the magnitude of the binding effect. Although, as discussed in the “Introduction”, there is some evidence to suggest that children privilege temporal cues more so than adults when making causal judgements (e.g., McCormack et al., 2015; Schlottmann, 1999), we found no evidence that children’s timing judgements are in turn more heavily influenced by their causal beliefs. In previous studies that indicated that children tended to privilege temporal cues in making causal judgements, such cues were pitted against statistical information or information about causal mechanism. One plausible reason that children privileged temporal information is because it is salient and easier to process and bring to bear in making causal judgements than these other types of information (see McCormack, 2015, for discussion). That is, perhaps temporal cues have a special status with regard to children’s causal judgements because making use of such cues is less costly in terms of processing resources (though see White, 2014, for an alternative explanation). However, it is not obvious that there is any similar saving in processing costs when temporal judgements are influenced by causal beliefs; given that, it is perhaps not surprising that larger binding effects were not observed in children than in adults. Nevertheless, the fact that temporal binding can be observed in young children, and does not increase in magnitude across development, strongly suggests that the bidirectionality of the relation between time and causation is fundamental.

In addition to understanding the developmental roots of the binding effect, exploration of the effect from a developmental perspective is of interest because measuring binding in the context of intentional action has been suggested as a way of implicitly measuring the sense that one is the cause of an outcome (sense of agency; e.g., Caspar et al., 2016, 2018). An implicit measure of sense of agency might be thought to be particularly useful in a developmental context, both because of the potential difficulties in asking children to make explicit judgements of sense of agency, and because of the possibility that sense of agency itself might show interesting developmental patterns (Metcalfe et al., 2010; van Elk et al., 2015). We note, though, that our findings in Experiment 2, along with other published findings (e.g., Buehner, 2012; Poonian & Cunnington, 2013; Suzuki et al., 2019), indicate that temporal binding is observed even when the cause of an outcome is not a self-generated action, suggesting that it is a broader effect stemming from causal representations rather than reflecting a sense of agency per se. Nevertheless, the development of appropriate ways of measuring binding in children may provide researchers with an alternative to explicit causal judgements, thus allowing for the assessment of causal cognition in young children who may be less able to explicitly articulate their causal knowledge. That is, future studies could potentially use the binding effect as an index of children’s causal representations.

## Conclusion

In conclusion, Experiment 1 presented a novel paradigm that was successfully used to elicit the binding effect in both adults and children. The results indicated that both adults and children were more likely to perceive delays between causes and effects as shorter than delays between unrelated events. Experiment 2 provided further evidence for the suitability of the new paradigm and expanded the results to show that the binding effect is not limited to self-action; it also occurs, albeit to a lesser extent, when observing the actions of others. That is, both adults and children showed greater binding for self-generated action-outcome sequences than for observed action–outcome sequences. Taken together, these results suggest that although causality underlies the effect in both children and adults, the addition of intentionality can modulate the magnitude of the effect. These experimental results present the first evidence that the binding effect is present and consistent from childhood into adulthood.
